# Hepatic glycogen storage diseases type 0, VI and IX: description of an italian cohort

**DOI:** 10.1186/s13023-022-02431-5

**Published:** 2022-07-19

**Authors:** Francesco Tagliaferri, Miriam Massese, Luisa Russo, Anna Commone, Serena Gasperini, Roberta Pretese, Carlo Dionisi-Vici, Arianna Maiorana

**Affiliations:** 1 Division of Metabolism, Department of Pediatric Subspecialties, Bambino Gesù Children’s Hospital, IRCCS, Rome, Italy; 2 SCDU of Pediatrics, Azienda Ospedaliero-Universitaria Maggiore Della Carità, University of Piemonte Orientale, Novara, Italy; 3 Center for Rare Diseases and Birth Defects, Department of Woman and Child Health and Public Health, Fondazione Policlinico Universitario A. Gemelli IRCCS, Rome, Italy; 4 Metabolic Unit Rare Disease, Pediatric Department, Fondazione MBBM, San Gerardo Hospital, Monza, Italy

**Keywords:** Glycogen storage disease, Liver, Nutrition, Nutritional therapy, Insulin-resistance, Overweight, Obesity

## Abstract

**Background:**

Glycogen storage disease (GSD) type 0, VI and IX are inborn errors of metabolism involving hepatic glycogen synthesis and degradation. We performed a characterization of a large Italian cohort of 30 patients with GSD type 0a, VI, IXa, IXb and IXc. A retrospective evaluation of genetical, auxological and endocrinological data, biochemical tests, and nutritional intakes was assessed. Eventual findings of overweight/obesity and insulin-resistance were correlated with diet composition.

**Results:**

Six GSD-0a, 1 GSD-VI, and 23 GSD-IX patients were enrolled, with an age of presentation from 0 to 72 months (median 14 months). Diagnosis was made at a median age of 30 months, with a median diagnostic delay of 11 months and a median follow-up of 66 months. From first to last visit, patients gained a median height of 0.6 SDS (from − 1.1 to 2.1 SDS) and a median weight of 0.5 SDS (from − 2.5 to 3.3 SDS); mean and minimal glucose values significant improved (*p* < 0.05). With respect to dietary intakes, protein intake (g/kg) and protein intake (g/kg)/RDA ratio directly correlated with the glucose/insulin ratio (*p* < 0.05) and inversely correlated with HOMA-IR (Homeostasis model assessment of insulin resistance, *p* < 0.05), BMI SDS (*p* < 0.05) and %ibw (ideal body weight percentage, *p* < 0.01).

**Conclusion:**

A prompt establishment of specific nutritional therapy allowed to preserve growth, improve glycemic control and prevent liver complication, during childhood. Remarkably, the administration of a high protein diet appeared to have a protective effect against overweight/obesity and insulin-resistance.

## Background

**Fig. 1:**
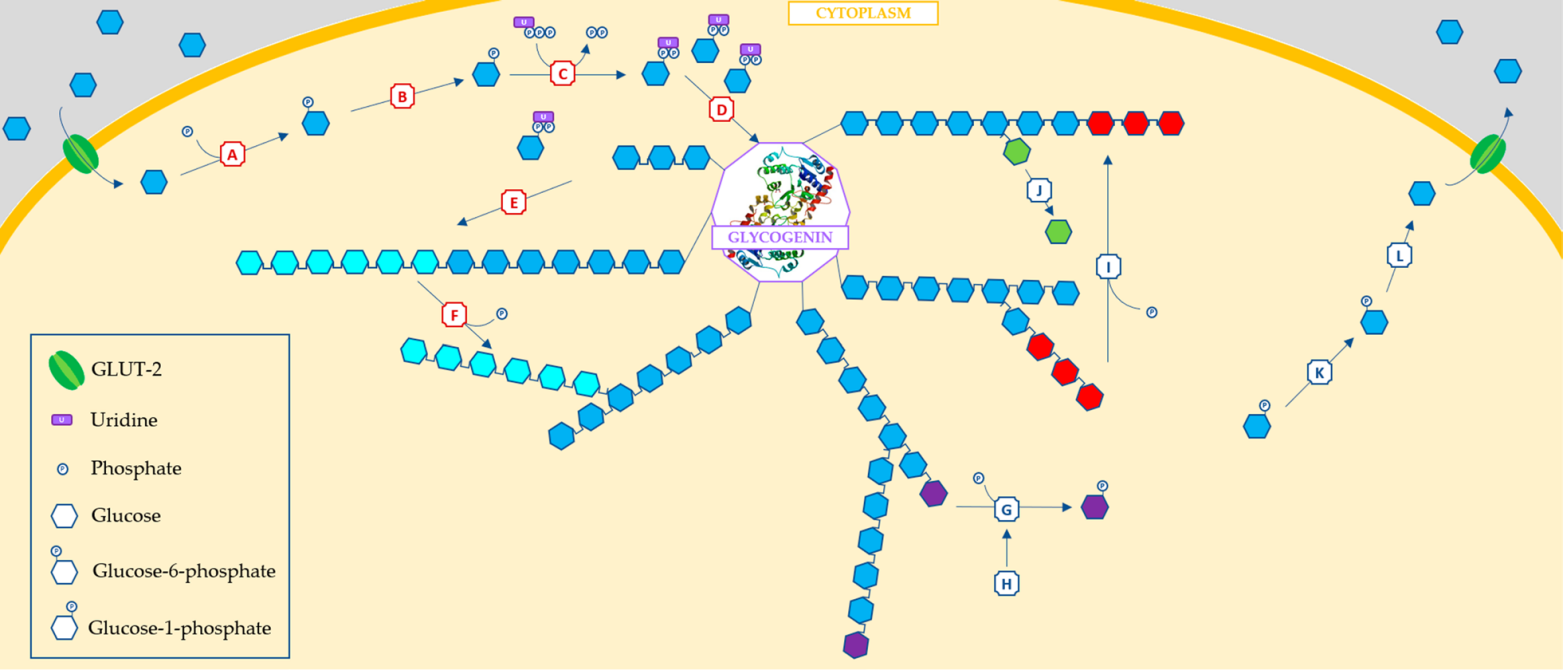
Main cytoplasmatic pathways involved in glycogen synthesis and breakdown in the liver cell.** A**, Glucokinase: phosphorylates glucose to glucose 6-phosphate;** B**, Phosphoglucomutase: shifts phosphate to create glucose 1-phosphate;** C**, UDP-glucose pyrophosphorylase: combines glucose 6-phosphate with UTP to uridine diphosphate;** D**, UDP-glucose:glycogenin glucosyltransferase: glycogenin catalyses its own glycosylation until a base of 5–13 glucose molecules;** E**, Glycogen synthase: incorporates glucose into glycogen via an α-1,4-glycosidic bond;** F**, Branching enzyme: every 10 to 14 glucose units, catalyses the shift of seven glucose molecules to a side branch (light blue), connected with an α-1,6-glycosidic bond;** G**, Glycogen phosphorylase: breaks peripheral α-1,4-glycosidic bond to release glucose 1-phosphate (purple);** H**, Glycogen phosphorylase kinase: phosphorylates glycogen phosphorylase triggering a conformational shift to a more active form;** I**, Debranching enzyme: when four molecules remain on a side chain, it transfers three of those to a primary chain (red);** J**, α-1,6-glucosidase: removes the last glucose from a side chain;** K**, Phosphoglucomutase: converts glucose 6-phosphate to glucose 1-phosphate;** L**, Glucose 6-phosphatase: removes phosphate group from glucose 6-phosphate

Glycogen is a highly branched polymer of glucose molecules. It is the main storage form of carbohydrate in humans, primarily within liver and muscles [1]. Its role is to store glucose and make it available as soon as glycaemia gets low. Glycogen formation and breakdown are strictly dependent on hormone regulation (insulin vs glucagon and epinephrine) and involve several enzymes (Fig. 1).

Starting from glycogenin, glucose molecules are added consecutively (α-1,4-glycosidic bonds, glycogen synthase) and partially shifted to form multiple ramifications (α-1,6-glycosidic bonds, branching enzyme) [2]. If necessary, having many free terminations allows glycogen to undergo the simultaneous action of different phosphorylases which, along with debranching enzyme and phosphoglucomutase, lead to a quick release of glucose. Hence, hepatic glycogen is a prompt source of glucose during a short period of fasting (3–4 h), while muscular one is used in early phase of exercise.

Glycogen storage diseases (GSDs) are inborn metabolic disorders caused by deficiency of enzymes involved in the synthesis or degradation of glycogen [3]; the main affected organs are the liver, muscle and heart [4]. Among the hepatic forms, we will focus on GSD-0a, GSD-VI and GSD-IX.

Liver glycogen synthase deficiency (Fig. 1, E) (OMIM 240600), also known as GSD-0a, is caused by mutations in the GYS2 gene (OMIM 138571), which encodes the hepatic isoform of glycogen synthase and is located on chromosome 12p12.2. GSD-0a is inherited in an autosomal recessive manner [5]. Fasting ketotic hypoglycemia accompanied by low levels of alanine and lactate is the main clinical feature, usually appearing for the first time in late infancy. Lethargy or hypoglycemic seizures may present during intercurrent illnesses. Short stature, failure to thrive, hyperlipidemia, or elevation of hepatic transaminase levels can be subtle manifestations [6]. The absence of the synthase prevents the accumulation of glycogen, therefore hepatomegaly will not usually occur [7]. After consumption of carbohydrates, inability to store glucose as glycogen in the liver results in postprandial hyperglycemia. Glucose and other sugars taken up by the liver in GSD-0a are shunted into the glycolytic pathway leading to postprandial hyperlactatemia. Furthermore, Acetyl CoA formation stimulates lipogenesis causing hyperlipidemia [6].

GSD-VI (also known as Hers disease, OMIM 232700) is the result of a deficiency of liver glycogen phosphorylase (Fig. 1, G). It is caused by mutations in PYGL gene (OMIM 613741), located on chromosome 14q21-q22.3 with an autosomal recessive inheritance [8]. The enzyme catalyzes the phosphorylytic cleavage of α-1,4-glycosidic bonds to release glucose 1-phosphate [9]. GSD-VI is usually a relatively mild disorder, presenting in early childhood with hepatomegaly, growth retardation, mild hypoglycemia, and ketosis [8]. However, some more severe phenotypes with pronounced hypoglycemia, marked hepatomegaly, liver adenomas, liver fibrosis, muscular hypotonia, and post-prandial lactic acid elevation have been described [10, 11]. Other biochemical features include elevated hepatic transaminases, hyperlipidemia and low prealbumin level [12].

GSD-IX, liver form (OMIM 306000), is caused by liver phosphorylase kinase (PhK) deficiency (Fig. 1, H). The enzyme is a tetramer composed of α, β, γ, and δ subunits [13]. Several genes contain the information for these proteins. The muscle α-subunit is encoded by the PHKA1 gene (Xq13.1; OMIM 311870), while PHKA2 (Xp22.13; OMIM 300798) codifies for the liver α-subunit [14]. They are both transmitted as X-recessive trait [11]. Mutations in PHKB (16q12.1; OMIM 172490) and PHKG2 (16p11.2; OMIM 172471) genes cause autosomal recessive forms of liver PhK deficiency [14], codifying for β and γ subunits respectively; muscle γ isoform is encoded by PHKG1 (OMIM 172470). Calcium levels regulate the effect of the enzyme via calmodulin, the δ-subunit. This part of PhK is encoded by three different genes—CALM1 (OMIM 114180), CALM2 (OMIM 114182), and CALM3 (OMIM 114183)—which are ubiquitously expressed and involved in other cellular processes [11]. Patients with the hepatic forms of GSD-IX carry pathogenic variants in the PHKA2, PHKB, and PHKG2 genes. Clinical phenotype usually overlaps GSD-VI features, making the two forms difficult to be distinguished without molecular analysis.

In this study, we characterized a large Italian cohort with GSD-0a, GSD-VI and GSD-IX with a total follow-up of 173 patient years (median: 5.5 years, min–max: 0.6–17 years), focusing on auxological and metabolic parameters and eventual signs of insulin-resistance.

## Results

### Age distribution

Six GSD-0a (two males and four females), 1 GSD-VI (one male), and 23 GSD-IX (nineteen males and four females) patients were enrolled. Among GSD-IX patients, 17 had GSD-IXa (X-linked form, all males), 4 had GSD-IXb form, 2 had GSD-IXc.

The age of presentation in all patients ranged from 0 to 72 months with a median value of 14 months. In our cohort, the diagnosis was made at a median age of 30 months, with a median diagnostic delay of 11 months (Table 1).

**Table 1:** Age of presentation, diagnosis, and diagnostic delay

	Onset		Diagnosis		Diagnostic delay	
Type (n)	Median (m)	IQR (m)	Median (m)	IQR (m)	Median (m)	IQR (m)
GSD-0a (6)	14.5	47.8	69.5	77.5	25	27.8
GSD-VI (1)	14	–	20	–	6	–
GSD-IX (23)	14	14.5	27	30.5	11	10.5
All (30)	14	15.3	30	38.8	11	14.3

Statistical analysis between each group did not reveal significant differences

IQR, interquartile range; m, months

The age at first visit in all patients ranged from 0.3 to 16.5 years with a median value of 2.3 years. A single patient was referred to Ospedale Pediatrico Bambino Gesù at 16.5 years. In the last visits, the age ranged from 1.8 to 19.6 years (median 8.8) (Table 2).

**Table 2:** Age of the first and last visits

Type (n)	First visit		Last visit	
	Median (y)	IQR (y)	Median (y)	IQR (y)
GSD-0a (6)	1.9	3.1	9.1	0.5
GSD-VI (1)	1.6	–	5.2	–
GSD-IX (23)	2.5	2.7	8.2	7.8
All (30)	2.3	2.7	8.8	6.2

IQR, interquartile range; y, years

### Genetic data

The complete list of genetic variants for each patient, together with the segregation data, is available in Table 3.

**Table 3:** Molecular analysis of the cohort

GSD type	Gene	Gender	Variants	Protein	Coding impact	ACMG/AMP classification	Inheritance
0a	GYS2 12p12.1	♂	c.574C > T	p.Arg192Ter	Nonsense	5	M
			c.574C > T	p.Arg192Ter	Nonsense	5	P
		♀	c.1322C > T	p.Pro441Leu	Missense	3	P*
			c.1400A > T	p.Asn467Ile	Missense	3	M
			c.1965G > C	p.Gln655His	Missense	1	P*
		♀	c.1156C > T	p.Arg386Ter	Nonsense	5	P
			c.1436C > A	p.Pro479Gln	Missense	4	M
		♀	C.163A > G	p.Thr55Ala	Missense	3	NA
			c.1169G > C	p.Trp390Ser	Missense	3	NA
		♀	c.1062 + 1G > T	p.?	Splicing	5	NA
			c.1965G > C	p.Gln655His	Missense	1	NA
		♂	c.736C > T	p.Arg246Ter	Nonsense	5	P
			c.1436C > A	p.Pro479Gln	Missense	4	M
VI	PYGL	♂	c.2 T > A	p.Met1Lys	Start loss	5	M
	17q21.31		c.1015A > G	p.Asn339Asp	Missense	3	P
IXa	PHKA2 Xp22.13	♂	c.133C > T	p.Arg45Trp	Missense	4	M
		♂	c.134G > A	p.Arg45Gln	Missense	4	M
		♂	c.328delG	p.Asp110ThrfsTer39	Frameshift	5	de novo
		♂	c.571A > T	p.Asn191Tyr	Missense	3	M
		♂	c.618G > A	p.Lys206 =	Synonimous, splice junction loss	5	M
		♂	c.928C > T	p.Arg310Ter	Nonsense	5	M
		♂	c.1166_1167delCA	p.Thr389SerfsTer33	Frameshift	5	M
		♂	c.2443G > A	p.Gly815Ser	Missense	3	M
		♂	c.2675A > G	p.Gln892Arg	Missense	5	M
		♂	c.2677-2A > G	p.?	Splicing	5	M
		♂	c.2746C > T	p.Arg916Trp	Missense	5	M
		♂	c.2746C > T	p.Arg916Trp	Missense	5	M
		♂	c.3373G > A	p.Glu1125Lys	Missense	4	M
		♂	c.3512C > T	p.Ala1171Val	Missense	4	M
		♂	c.3614C > T	p.Pro1205Leu	Missense	5	M
		♂	Del206kb reg Xp22.13	p.?	Deletion	5	M (siblings)
		♂	Del206kb reg Xp22.13	p.?	Deletion	5	
IXb	PHKB 16q12.1	♂	c.511C > T	p.Gln171Ter	Nonsense	5	M
			c.1969C > T	p.Gln657Ter	Nonsense	5	P
		♀	c.1969C > T	p.Gln657Ter	Nonsense	5	M
			c.1969C > T	p.Gln657Ter	Nonsense	5	**
		♀	c.2275delG	p.Glu759LysfsTer38	Frameshift	5	M
			c.2275delG	p.Glu759LysfsTer38	Frameshift	5	F
		♀	c.2536G > T	p.Glu846Ter	Nonsense	5	NA
			c.2536G > T	p.Glu846Ter	Nonsense	5	NA
IXc	PHKG2 16p11.2	♂	c.112G > A	p.Val38Ile	Missense	5	M
			c.112G > A	p.Val38Ile	Missense	5	**
		♀	c.1070 T > C	p.Leu357Pro	Missense	4	M
			c.1070 T > C	p.Leu357Pro	Missense	4	P

ACMG/AMP classification: 5, Pathogenic; 4, Probably pathogenic; 3, Variant of uncertain significance (VUS); 2, Probably benign; 1, Benign. Inheritance: M, maternal; P, paternal; NA, not available

^*^cis

^**^Father not available, but consanguineous parents

### Anthropometric measures

Median height gain between first and last visit was 0.64 SDS (range from − 1.1 to 2.1). Patients manifested an increased median weight gain of 0.5 SDS (from − 2.5 to 3.3). Considering SDS gain of weight-for-length and BMI as a continuum, the overall cohort median value of 28 patients, for which data were available, was 0.2 SDS (from − 1.7 to 3.1). However, in patients below 2 years of age, the comparison between SDS weight-for-length at first visit and SDS BMI at last visit showed a median gain of 1.0 SDS (from − 1.7 to 3.1), while patients older than 2 years presented a median gain of 0.10 SDS (from − 1.7 to 1.3): the body mass gain between the two groups resulted significantly different (*p* < 0.05). GSD type subgroups analysis (GSD-0a vs GSD-VI/IX) for variations in BMI SDS and % ideal body weight (%ibw) did not show significant differences.

### Biochemical features

A significant rise in haemoglobin, plasma glucose, HDL cholesterol, insulin, IGF-1 and bicarbonate level was observed between first and last visit. Conversely, triglycerides and transaminases significantly reduced. The alkaline phosphatase, gammaGT and alpha fetoprotein levels also decreased. However, many patients at first visit were infants with physiological higher levels of these latter parameters (Table 4).

**Table 4:** Biochemical features

Blood test	First visit		Last visit		*p* value
	Median	IQR	Median	IQR	
Hb (mg/dL)	11.7	1.4	13.1	1.9	< 0.001
Glucose (mg/dL)	63	29.5	80	14.5	< 0.05
INR	1.1	0.1	1.1	0.1	ns
aPTT-ratio	0.9	0.1	1	0.1	ns
Fibrinogen (mg/dL)	289	56	286.5	89.3	ns
Triglycerides (mg/dL)	132.5	106	91	55	< 0.05
Total cholesterol (mg/dL)	155	42	155	46.5	ns
HDL (mg/dL)	27	9.3	37	15	< 0.05
LDL (mg/dL)	105	23.8	96	40	ns
Uric acid (mg/dL)	4.7	1.5	4	1.6	ns
NEFA (μM/L)	900.5	1020.5	745	472.5	ns
ALT (U/L)	96	194.8	31	57	< 0.05
AST (U/L)	83	188.8	36	45	< 0.01
CPK (U/L)	88.5	45	101	41	ns
LDH (U/L)	536	214	208	64.5	< 0.001
CHE (U/L)	7416	3482.5	8133	2293.5	ns
ALP (U/L)	586	466.5	264	177	< 0.001
GammaGT (U/L)	20	25	16	9	< 0.05
Alpha fetoprotein (ng/mL)	3.5	7.7	1.5	2	< 0.05
Basal insulin (µU/mL)	1.1	1.2	4.7	7.3	< 0.01
TSH (µU/mL)	2.7	1.9	3.3	1.2	ns
FT4 (ng/dL)	1.2	0.2	1.3	0.2	ns
IGF1 (ng/mL)	29	45.4	97.9	106.3	< 0.001
pH	7.36	0.03	7.4	0.03	ns
HCO3 (mmol/L)	20	3.5	22.7	1.8	< 0.001
ABE (mmol/L)	-4.8	3.4	-1.9	3	< 0.001
Lactate (mmol/L)	1.4	1	1.4	0.7	ns

IQR, interquartile range; ns, not significant

### Hepatic involvement

Hepatomegaly was present in 23 out of 30 (77%) patients (GSD-0a 2/6, GSD-VI 1/1, GSD-IX 20/23), while abdomen ultrasound revealed one more GSD-IXa patient with increased liver dimensions (24/30, 80%). Liver hyperechogenicity was found in 26/30 patients (87%), without nodular formations or cirrhosis. Seven patients underwent liver biopsy (23%) for diagnostic purpose. These patients were diagnosed before Next Generation Sequencing (NGS) technology advent.

### Glucose homeostasis

Almost half of the patients presented hypoglycemia in the first year of life. Assessment of glycemic control by 72-h continuous glucose monitoring system (CGMS) showed a significant improvement of mean and minimal glucose values (*p* < 0.05) from first to last visit. HbA1c and fructosamine were in the normal range at last visit; statistical comparisons were not possible due to lack of first visit data in most patients.

### Insulin-resistance parameters

**Fig. 2:**
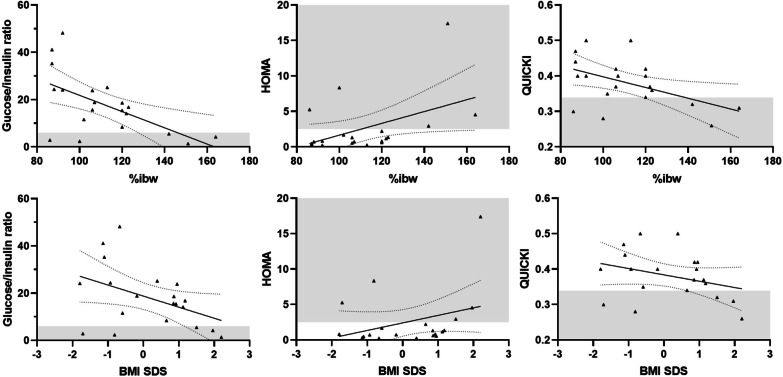
Correlations between insulin-resistance parameters, BMI SDS and %ibw. Glucose/insulin ratio and QUICKI inversely correlated with %ibw (upper row) and BMI SDS (lower row); HOMA index directly correlated with %ibw (upper row) and BMI SDS (lower row). The grey areas indicate normal values

Five patients of the overall cohort showed insulin-resistance defined as glucose/insulin ratio < 6 and/or Homeostasis model assessment of insulin resistance (HOMA-IR index) > 2.5. Between these patients, two showed obesity, one overweight and two were normal-weighted. Insulin-resistance parameters were correlated with anthropometric measures at last visit. We observed a significant inverse correlation between glucose/insulin ratio and %ibw (*p* < 0.001); an inverse trend of correlation was found with BMI SDS. HOMA index positively correlate with %ibw, significantly (*p* < 0.05), with a positive trend of correlation with BMI SDS. Accordingly, the insulin-sensitivity QUICKI (Quantitative insulin sensitivity check index), inversely correlated with %ibw significantly (*p* < 0.05) and with BMI SDS, although not significantly (Fig. 2). No differences in GSD type subgroups analysis (GSD-0a vs GSD-VI/IX) were found in glucose/insulin ratio, HOMA or QUICKI versus anthropometric measures (Fig. 2).

### Nutritional assessment

Irrespectively of dietary prescription, dietary recalls were used to evaluate the effective nutritional intakes. Table 5 shows the intakes at the first and last visit according to nutrients distribution: a significant rise in protein percentage was recorded. A significant increase of the total calories as well as a significant reduction of the total calories to recommended daily allowance (RDA) ratio, a significant decrease of the calories per kilogram and a significant decrease of glucose/kg/min intake reflected the normal pattern of nutrition during growth.

**Table 5:** Dietary intakes at first and last visits

	First visit		Last visit		*p* value
	Median	IQR	Median	IQR	
Carbohydrate%	58.4	10.1	57.1	11.1	ns
Lipid%	27.8	4.8	25.0	8.5	ns
Protein%	14.0	4.7	19.1	6.4	< 0.001
Carbohydrate%/RDA ratio	1.1	0.2	1.1	0.2	ns
Lipid%/RDA ratio	0.8	0.3	0.9	0.3	ns
Protein%/RDA ratio	1.2	0.7	1.0	0.4	ns
Protein (g/kg)	2.9	1.7	2.3	1.2	ns
Protein (g/kg)/RDA ratio	2.8	1.6	2.3	1.2	ns
Total calories	1079.0	312.3	1686.7	574.3	< 0.001
Calories/RDA ratio	1.0	0.4	0.8	0.3	< 0.05
Calories/kg	76.2	37.5	54.3	45.0	< 0.001
Glucose mg/kg/min	7.4	4.7	4.7	4.0	< 0.01

IQR, interquartile range; ns, not significant

The intakes at last visit were correlated with body mass and insulin resistance indices. The percentages of the various macronutrients and the RDA ratios did not significantly correlate with any data. Conversely, the protein intake (g/kg) and its RDA ratio directly correlated with the glucose/insulin ratio (*p* < 0.05) and inversely correlated with HOMA (*p* < 0.05), BMI SDS (*p* < 0.05) and %ibw (*p* < 0.01). Finally, the amount of calories, adjusted for the recommended requirement for age, did not correlate with any of the outlined parameters.

## Discussion

We reported a large cohort of patients with GSD-0a, GSD-VI and GSD-IX from two Italian centres. GSD-IX patients account for the 77% of the cohort (23 patients), while GSD-0a was less represented (20%, 6 patients). Only 1 patient with GSD-VI was present (3%). Since the subgroup analysis did not reveal any difference, we considered the overall cohort for statistical analysis.

The liver involvement, hallmark of the disease, was present in 77% of our patients as hepatomegaly at physical examination. Enlarged liver was found in two out of six GSD-0a patients, consistently with recent reports [15, 16]. Abdomen ultrasound allowed to confirm the clinical finding and to characterize the increased echogenicity, as well as to monitor the liver evolution over time: for this reason, it is recommended to perform it in patients with hepatic GSD once every 12–24 months [11]. In our cohort, only minimal changes were observed during follow-up in GSD-IX patients: hepatomegaly resolved in 2 patients, and hyperechogenicity improved in one patient. Liver cirrhosis was not present in our cohort, even though previously reported in the literature [9, 17, 18].

During the diagnostic process, 7 out of 30 patients underwent liver biopsy, equal to 23.3%: this approach in the last years has been sidelined by the improvement in genetic NGS technology, which is less invasive and allows family screening in addition to diagnosis [8]. In our cohort, every patient has been genetically tested. All mutations were found on the Varsome search engine [19]. In some patients presenting a phenotype compatible with the diagnosis, after exclusion of other carbohydrate disorders, variants of uncertain significance (VUS) were considered as potential disease-causing, according to the clinical and biochemical presentation. Furthermore, in one patient with GSD-0a the mutation c.1965G > C, classified as VUS at the time of diagnosis, was reclassified as benign a few years later. Similarly, other variants might be reclassified over time, as genetic databases are progressively updated with the evolution of knowledge.

Once GSD-0a, GSD-VI and GSD-IX have been diagnosed, patients were instructed to avoid fasting, eating frequent protein-rich meals [20]. The increase in protein intake exploits gluconeogenesis for glucose and energy generation, thus reducing the glycogen storage in liver and muscle [11]. Uncooked cornstarch administration was also recommended in patients who experienced hypoglycemia [20]. The protein intake of our patients at the last visit increased by approximately 50% compared to the first visit. The percentage of protein intake was not significantly correlated with insulin-resistance indices. However, a significant direct correlation between the protein intake (g/kg) and the RDA ratio with the glucose/insulin ratio was found. On the contrary, a significant inverse correlation between protein intake (g/kg) and the RDA ratio with HOMA, BMI and %ibw was recorded. In turn, an inverse correlation between BMI and %ibw with glucose/insulin ratio and QUICKI and a direct correlation with HOMA index were found, indicating a linear correlation between body mass gain and insulin-resistance development. These findings suggest a protective effect of protein rich meals against overweight/obesity and insulin-resistance in GSD patients. Speculatively, these correlations may be explained by the lipolytic effect of a high-protein diet [21] and by its ameliorating effect on insulin-sensitivity [22].

BMI SDSs were used to assess the prevalence of overweight and obesity in our cohort at first and last examination. Although not significant, overweight (as BMI SDS > 1.04) and obese (as SDS > 1.64) patients’ prevalence increased (Table 6). The weight gain was more relevant after the COVID-19 lockdown, likely due to the restriction of physical activity [23]. Interestingly, patients younger than 2 years at the first evaluation had a median body mass SDS gain greater than older ones.

**Table 6:** Prevalence of overweight and obese at last visit in patients who had the first evaluation before and after 2 years of age

Age at first visit (n)	First visit		Last visit	
	Overweight (%)	Obese (%)	Overweight (%)	Obese (%)
< 2 years (12)	2–16.7	1–8.3	6–50.0	4–33.3
> 2 years (16)	4–25.0	1–6.3	5–31.3	1–6.3
All (28)	6–21.4	2–7.1	11–39.3	5–17.9

This finding was in agreement with the more pronounced increase in overweight in this group at last visit (50% vs 16.7%). It could be speculated that, due to the higher prevalence of hypoglycemia in the younger patients, the nutritional modifications, consisting in more hyperglucidic and hypercaloric diets in the first years of age, could have led to increase the overweight.

Considering %ibw at last visit, 16 patients resulted overweight (> 110%), 13 with mild obesity (> 120%), 5 with moderate obesity (> 140%) and 2 were in severe obesity range (> 160%). However, the %ibw in pediatric patients is reported to be not so accurate [24].

Biochemical analyses allowed to detect a significant improvement between first and last visits, primarily in lipid profile (reduction of triglycerides, increase of HDL) and glycemic control (rise of mean and minimal values of glycemia). Moreover, the decrease in transaminases witnessed the hepatic improvement.

Growth retardation is another feature of the disease: 5 out of 28 of our patients had an SDS height lower than − 2 SDS at the first visit, equal to about 18%. At the last evaluation, this percentage lowered up to 7%, indicating an improvement also in linear growth, according to what reported for PhK-deficient patients [25], who were the most represented in our cohort.

We are aware of some limitations. We retrospectively collected patients’ data from first and last evaluations: some data were missing at first visits, not allowing the comparison of some parameters. Segregation studies were performed in majority of parents, but they were not available in 3 samples. Furthermore, due to the heterogeneity of the cohort, age and follow-up duration varied consistently. To cope with that, data were normalized to percentiles, minimizing the comparison bias.

In conclusion, we described a large cohort of GSD-0a, GSD-VI and GSD-IX patients. A prompt establishment of specific nutritional therapy allowed to preserve growth, improve glycemic control and prevent liver complication, during childhood. Patients manifested a body mass gain after a median follow-up of 5.5 years, especially in those diagnosed before the age of 2 years, when the diet is likely more hyperglucidic and hypercaloric. The administration of a high protein diet appeared to have a protective effect against overweight/obesity and insulin-resistance. Future collaborative and longitudinal studies may allow a better knowledge of the diseases and improve the management of patients.

## Methods

### Patients

A total of 30 patients with diagnosis of glycogen storage disease type 0, VI, IXa, IXb and IXc were included in the study. Patients were collected from two Italian Metabolic Disease Centers, the Ospedale Pediatrico Bambino Gesù of Rome, and the San Gerardo Hospital of Monza. Patients’ data were retrospectively collected and analyzed from clinical reports of the first and last available visits. The study was conducted in accordance with the Declaration of Helsinki. Ethical standards and informed consent were obtained from parents. Ethical approval was waived for this study because it was a retrospective clinical study.

### Auxological parameters

Length and standing height (for patient younger and older than 2 years, respectively) and weights were assessed. Standing heights were measured by wall-mounted stadiometer to the nearest 0.1 cm; weight was measured to the nearest 0.1 kg. SIEDP Growth 4.0® by Eli Lilly was used to derive height and weight percentiles and SDS, Z-scores. The CDC 2002 growth charts below 2 years of age and the Italian cross sectional growth charts [26] above 2 years of age were used. BMI and weight/length ratio SDS have been derived for patient older and younger than 2 years respectively. Lastly, we calculated the ideal body weight percentage (%ibw) using the Traub method [27].

### Nutritional parameters

Dietary recalls were used to collect information on carbohydrates, proteins, and lipids in grams per day, as well as the total calories count. Hence, we calculated the corresponding percentage of calories provided by each category of nutrients and the glucose mg/kg/min intake. These data were finally compared with RDA indications.

### Biochemical parameters

Blood tests were collected to assess organ involvement. Particularly, we focused on hemoglobin, glucose, coagulation (INR, aPTT-ratio, fibrinogen), lipid profile (triglycerides, total cholesterol, HDL, LDL), uric acid, NEFA, ALT, AST, GGT, CPK, LDH, CHE, ALP, alpha fetoprotein, basal insulin, TSH and FT4, IGF1, acid–base assessment (pH, HCO3, ABE), and lactate.

### Insulin-resistance parameters

In patients with available fasting plasma insulin and glucose measurements, insulin-resistance indexes were calculated: glycemia [mg/dl]/insulin [μU/mL] ratio; Homeostasis model assessment of insulin resistance (HOMA-IR) index was calculated as follows: (fasting plasma glucose (FPG) [mmol/L] × fasting insulin μU/mL)/22.5; Quantitative insulin sensitivity check index (QUICKI) was calculated as follows: 1/[log fasting insulin μU/mL + log FPG mg/dL].

### Genetic analysis

Molecular analysis was performed for each patient using Sanger sequencing and NGS gene panel for GSDs in more recent years. Other techniques were applied to complete the genetic study in cases of negative or partial results, including high-resolution CGH-Array. Through Varsome as search engine [19], individual variants were characterized using coding impact and the American College of Medical Genetics and Genomics, and the Association for Molecular Pathology (ACMG/AMP) guidelines for the interpretation of sequence variants [28].

### Statistics

Statistical analysis was performed with Stata® (ver.14.1 SE, Stata Corporation, College Station, TX, USA). Wilcoxon signed-rank test and Mann–Whitney *U* test were utilized, as appropriate. Correlation study was performed by Spearman’s rank correlation. Statistical significance was set at *p* < 0.05.

## Data Availability

Data can be shared upon request, contacting the corresponding author Dr. Arianna Maiorana.
